# Supplementary material for: Impact of Food-Based Weight Loss Interventions on Gut Microbiome in Individuals with Obesity: A Systematic Review
Source: Nutrients. 2022 May 6;14(9):1953. doi: 10.3390/nu14091953 (PMC9099876; doi:10.3390/nu14091953)
Supplement: Supplementary file 1 [file nutrients-14-01953-s001.zip › nutrients-1669016-supplementary.pdf]

Supplementary Materials

Table S1. Search strategy

Database	Search Strategy
Embase	('body weight loss'/de OR 'weight loss':ti,ab OR 'weight reduction':ti,ab OR 'bmi reduction':ti,ab OR 'bmi loss':ti,ab) AND ('microflora'/exp OR microbiota:ti,ab OR microbiome*:ti,ab) AND ('obesity'/de OR 'abdominal obesity'/exp OR 'morbid obesity'/exp OR overweight:ti,ab OR obese:ti,ab OR obesity:ti,ab) NOT ('child'/exp NOT 'adult'/exp)
Scopus	((INDEXTERMS("Weight Loss") OR (TITLE-ABS("Weight Loss") OR TITLE-ABS("weight reduction") OR TITLE-ABS("bmi reduction") OR TITLE-ABS("bmi loss"))) AND (INDEXTERMS("Microbiota") OR (TITLE-ABS("Microbiota") OR TITLE-ABS("microbiome*"))) AND (INDEXTERMS("Overweight") OR INDEXTERMS("Obesity") OR INDEXTERMS("obesity, morbid") OR (TITLE-ABS("Overweight") OR TITLE-ABS("obese") OR TITLE-ABS("Obesity")))) AND NOT (INDEXTERMS("child") AND NOT INDEXTERMS("adult"))
PubMed	(("Weight Loss"[MeSH Terms:noexp] OR ("Weight Loss"[Title/Abstract] OR "weight reduction"[Title/Abstract] OR "bmi reduction"[Title/Abstract] OR "bmi loss"[Title/Abstract])) AND ("Microbiota"[MeSH Terms] OR ("Microbiota"[Title/Abstract] OR "microbiome*"[Title/Abstract])) AND ("Overweight"[MeSH Terms:noexp] OR "Obesity"[MeSH Terms:noexp] OR "obesity, morbid"[MeSH Terms] OR ("Overweight"[Title/Abstract] OR "obese"[Title/Abstract] OR "Obesity"[Title/Abstract]))) NOT ("child"[MeSH Terms] NOT "adult"[MeSH Terms])
CINAHL	(((MH "Weight Loss") OR (TI "Weight Loss" OR AB "Weight Loss") OR (TI "weight reduction" OR AB "weight reduction") OR (TI "bmi reduction" OR AB "bmi reduction") OR (TI "bmi loss" OR AB "bmi loss")) AND ((MH "Microbiota+") OR (TI Microbiota OR AB Microbiota) OR (TI microbiome* OR AB microbiome*)) AND ((MH "Obesity") OR (MH "Obesity, Morbid") OR (TI Overweight OR AB Overweight) OR (TI obese OR AB obese) OR (TI Obesity OR AB Obesity))) NOT ((MH "Child+") NOT (MH "Adult+"))

Table S2. Reasons for excluding studies following full-text assessment*

Study citation	Reason for exclusion
Alemán 2018 [1]	Not an intervention of interest (VLCD)
Allen 2017 [2]	Abstract only
Basciani 2020 [3]	Not an intervention of interest (VLCKD)
Benítez-Páez 2021 [4]	Duplicate
Christensen 2019 [5]	Not an intervention of interest (no caloric restriction)
Cuevas Sierra 2020 [6]	Abstract only
Damms-Machado 2015 [7]	Not an intervention of interest (VLCD)
Dao 2018 [8]	Not an outcome of interest (no pre vs post microbiome data reported)
Di Rosa 2020 [9]	Not an intervention of interest (VLCKD)
Diener 2021 [10]	Macronutrient intake not reported
Dong 2021 [11]	Not an outcome of interest (no pre vs post microbiome data reported)
Duncan 2007 [12]	No weight loss achieved
Fabian 2017 [13]	Abstract only
Fangmann 2016 [14]	Not an intervention of interest (VLCD)
Gabel 2020 [15]	Abstract only
Gabel 2020 [16]	Macronutrient intake not reported
Gardner 2017 [17]	Not an outcome of interest (no pre vs post microbiome data reported)
Gratz 2020 [18]	Abstract only
Grembi 2020 [19]	Not an outcome of interest (no pre vs post microbiome data reported)
Heianza 2017 [20]	Not an outcome of interest (no microbiome or SCFA data)
Heianza 2018 [21]	Not an outcome of interest (no pre vs post microbiome data reported)
Heinsen 2016 [22]	Not an intervention of interest (VLCD)
Henning 2019 [23]	Not an intervention of interest (used a single food (avocado) only)
Hjorth 2019 [24]	Not an outcome of interest (no pre vs post microbiome data reported)
Hjorth 2020 [25]	Not a healthy population
Holt 2016 [26]	Not an intervention of interest (VLCD)
Hric 2021 [27]	Participants not overweight
Jie 2021 [28]	Not an outcome of interest (no pre vs post microbiome data reported)
Kahleova 2021 [29]	Abstract only
Karl 2015 [30]	Not an outcome of interest (no pre vs post microbiome data reported)
Kayser 2019 [31]	Not an outcome of interest (no pre vs post microbiome data reported)
Kong 2011 [32]	Abstract only
Kong 2011 [33]	Abstract only
Lee 2016 [34]	Not a healthy population
Li 2019 [35]	Abstract only
Lin 2018 [36]	Abstract only
Lin 2019 [37]	Macronutrient intake not reported
Kong 2013 [38]	Duplicate
Kong 2013 [38]	Not an outcome of interest (no pre vs post microbiome data reported)
Louis 2016 [39]	Not an intervention of interest (VLCD)

Table S2. continued

Study citation	Reason for exclusion
MacHado 2012 [40]	Not an intervention of interest (VLCD)
Mahadzir 2017 [41]	Not an intervention of interest (probiotic supplement only)
Mueller 2020 [42]	Not a healthy population
Muñiz-Pedrogo 2016 [43]	Abstract only
Muñiz-Pedrogo 2018 [44]	Macronutrient intake not reported
Muralidharan 2021 [45]	Not a healthy population
Nogacka 2020 [46]	Duplicate
Oh 2016 [47]	Case study
Ott 2017 [48]	Not an intervention of interest (VLCD)
Reimer 2017 [49]	Not an intervention of interest (no caloric restriction)
Remely 2015 [50]	Macronutrient intake not reported
Rinott 2021 [51]	Not an outcome of interest (no pre vs post microbiome data reported)
Roager 2019 [52]	No weight loss achieved
Russell 2011 [53]	No weight loss achieved
Salonen 2014 [54]	No weight loss achieved
Salonen 2014 [54]	Duplicate
Sánchez Alcoholado 2019 [55]	Abstract only
Siebert 2021 [56]	Not an outcome of interest (no pre vs post microbiome data reported)
Simões 2014 [57]	Not an intervention of interest (VLCD)
von Schwartzenberg 2021 [58]	Not an intervention of interest (VLCD)
Yoriko 2018 [59]	Not an outcome of interest (no microbiome or SCFA data)
Yuan 2021 [60]	Not an intervention of interest (ketogenic diet)
Zhou 2019 [61]	Not an outcome of interest (no microbiome or SCFA data)
Zolotarevsky 2018 [62]	Not a healthy population
Zou 2020 [63]	Participants not overweight

* Citation numbers do not correspond to citations in the main manuscript and are provided at the end of this document. VLCD: very low calorie diet; VLCKD: very low calorie ketogenic diet.

	D1	D2	D3	D4	D5	D6	D7
Bendsten 2018	?	?	+	+	+	?	+
Benítez-Páez 2021	?	?	+	+	+	?	+
Cuevas-Sierra 2021	+	?	+	+	+	?	+
Dhakal 2020	N/A	N/A	+	+	+	?	+
Dong 2020	+	?	+	+	+	?	+
Duncan 2008	?	?	+	+	+	?	+
Fragiadakis 2020	+	?	+	+	+	?	+
Gratz 2019	?	?	+	+	+	?	+
Gutiérrez-Repiso 2021	N/A	N/A	+	+	+	?	+
Jaagura 2021	N/A	N/A	+	+	+	?	+
Johnstone 2020	+	?	+	+	+	?	+
Kahleova 2020	+	?	+	+	+	?	+
Kahleova 2021	?	?	+	+	+	?	+
Ley 2006	?	?	+	+	+	?	+
Ma 2021	?	?	+	+	+	?	+
Nogacka 2021	N/A	N/A	+	+	+	?	+
Pisanu 2020	N/A	N/A	+	+	+	?	+
Stanislawksi 2021	+	?	+	+	+	?	+
Zhang 2021	+	+	+	+	+	?	+

Domains

D1: Sequence generation
D2: Allocation concealment
D3: Blinding of participants and personnel
D4: Blinding of outcome assessors
D5: Incomplete outcome data
D6: Selective outcome reporting
D7: Other sources of bias

Key

+ Low risk of bias
? Unclear risk of bias
− High risk of bias

Figure S1. Risk of bias assessment. N/A: not applicable (trial was not randomized).

References to Table S2 Citations

1. Alemán, J.O.; Bokulich, N.A.; Swann, J.R.; Walker, J.M.; Rosa, J.C.; Battaglia, T.; Costabile, A.; Pechlivanis, A.; Liang, Y.; Breslow, J.L.; et al. Fecal microbiota and bile acid interactions with systemic and adipose tissue metabolism in diet-induced weight loss of obese postmenopausal women. *J. Transl. Med.* **2018**, *16*, 244. https://doi.org/10.1186/s12967-018-1619-z.
2. Allen, J.M.; Nehra, V.; Mailing, L.; Holscher, H.D.; Van Dyke, C.T.; Edens, K.; Swanson, K.S.; Boardman, L.A.; Jensen, M.D.; Murray, J.A.; et al. Gut microbiota: Predictor of success in a comprehensive of lifestyle modification program for obesity. *Gastroenterology* **2017**, *152*, S626–S627.
3. Basciani, S.; Camajani, E.; Contini, S.; Persichetti, A.; Risi, R.; Bertoldi, L.; Strigari, L.; Prossomariti, G.; Watanabe, M.; Watanabe, S.; et al. Very-Low-Calorie Ketogenic Diets with Whey, Vegetable, or Animal Protein in Patients With Obesity: A Randomized Pilot Study. *J. Clin. Endocrinol. Metab.* **2020**, *105*, 2939–2949. https://doi.org/10.1210/clinem/dgaa336.
4. Benítez-Páez, A.; Hess, A.L.; Krautbauer, S.; Liebisch, G.; Christensen, L.; Hjorth, M.F.; Larsen, T.M.; Sanz, Y. Sex, Food, and the Gut Microbiota: Disparate Response to Caloric Restriction Diet with Fibre Supplementation in Women and Men. *Mol. Nutr. Food Res.* **2021**, *65*, e2000996. https://doi.org/10.1002/mnfr.202000996.
5. Christensen, L.; Vuholm, S.; Roager, H.M.; Nielsen, D.S.; Krych, L.; Kristensen, M.; Astrup, A.; Hjorth, M.F. Prevotella Abundance Predicts Weight Loss Success in Healthy, Overweight Adults Consuming a Whole-Grain Diet Ad Libitum: A Post Hoc Analysis of a 6-Wk Randomized Controlled Trial. *J. Nutr.* **2019**, *149*, 2174–2181. https://doi.org/10.1093/jn/nxz198.
6. Cuevas Sierra, A.; Riezu Boj, J.; Guruceaga, E.; Barceló, A.; Cuervo, M.; Milagro, F.; Martínez, A. Metagenomic biomarker in precision treatment for obesity: Microbiota composition change between before and after a nutrition intervention. *Obes. Rev.* **2020**, *21*, e13115. https://doi.org/10.1111/obr.13115.
7. Damms-Machado, A.; Mitra, S.; Schollenberger, A.E.; Kramer, K.M.; Meile, T.; Königsrainer, A.; Huson, D.H.; Bischoff, S.C. Effects of surgical and dietary weight loss therapy for obesity on gut microbiota composition and nutrient absorption. *Biomed. Res. Int.* **2015**, *2015*, 806248. https://doi.org/10.1155/2015/806248.
8. Dao, M.C.; Sokolovska, N.; Brazeilles, R.; Affeldt, S.; Pelloux, V.; Prifti, E.; Chilloux, J.; Verger, E.O.; Kayser, B.D.; Aron-Wisnewsky, J.; et al. A Data Integration Multi-Omics Approach to Study Calorie Restriction-Induced Changes in Insulin Sensitivity. *Front. Physiol.* **2018**, *9*, 1958. https://doi.org/10.3389/fphys.2018.01958.
9. Di Rosa, C.; Lattanzi, G.; Taylor, S.F.; Manfrini, S.; Khazrai, Y.M. Very low calorie ketogenic diets in overweight and obesity treatment: Effects on anthropometric parameters, body composition, satiety, lipid profile and microbiota. *Obes. Res. Clin. Pract.* **2020**, *14*, 491–503. https://doi.org/10.1016/j.orcp.2020.08.009.
10. Diener, C.; Qin, S.; Zhou, Y.; Patwardhan, S.; Tang, L.; Lovejoy, J.C.; Magis, A.T.; Price, N.D.; Hood, L.; Gibbons, S.M. Baseline Gut Metagenomic Functional Gene Signature Associated with Variable Weight Loss Responses following a Healthy Lifestyle Intervention in Humans. *mSystems* **2021**, *6*, e00964-21. https://doi.org/10.1128/mSystems.00964-21.
11. Dong, T.S.; Luu, K.; Lagishetty, V.; Sedighian, F.; Woo, S.L.; Dreskin, B.W.; Katzka, W.; Chang, C.; Zhou, Y.; Arias-Jayo, N.; et al. The Intestinal Microbiome Predicts Weight Loss on a Calorie-Restricted Diet and Is Associated With Improved Hepatic Steatosis. *Front. Nutr.* **2021**, *8*, 718661. https://doi.org/10.3389/fnut.2021.718661.
12. Duncan, S.H.; Belenguer, A.; Holtrop, G.; Johnstone, A.M.; Flint, H.J.; Lobley, G.E. Reduced dietary intake of carbohydrates by obese subjects results in decreased concentrations of butyrate and butyrate-producing bacteria in feces. *Appl. Environ. Microbiol.* **2007**, *73*, 1073–1078. https://doi.org/10.1128/AEM.02340-06.
13. Fabian, C.J.; Kimler, B.F.; Umar, S.; Ahmed, I.; Befort, C.A.; Nydegger, J.L.; Kreutzjans, A.L.; Powers, K.R.; Klemp, J.R.; Spaeth, K.R.; et al. Changes in the gut microbiome of post-menopausal women 2 weeks after initiating a structured weight loss intervention. *Cancer Res.* **2017**, *77*, P4-13-03. https://doi.org/10.1158/1538-7445.SABCS16-P4-13-03.
14. Fangmann, D.; Heinsen, F.A.; Schulte, D.M.; Rühlemann, M.C.; Türk, K.; Settgast, U.; Müller, N.; Lieb, W.; Baines, J.F.; Schreiber, S.; et al. Dietary and weight loss effects on human gut microbiome diversity and metabolism. *Diabetol. Stoffwechs.* **2016**, *11*, P166. https://doi.org/10.1055/s-0036-1580913.
15. Gabel, K.; Marcell, J.; Cares, K.; Kalam, F.; Cienfuegos, S.; Ezpeleta, M.; Varady, K. Effect of time restricted eating on the gut microbiome in adults with obesity. *Obesity* **2020**, *28*, 87. https://doi.org/10.1002/oby.23063.
16. Gabel, K.; Marcell, J.; Cares, K.; Kalam, F.; Cienfuegos, S.; Ezpeleta, M.; Varady, K.A. Effect of time restricted feeding on the gut microbiome in adults with obesity: A pilot study. *Nutr. Health* **2020**, *26*, 79–85. https://doi.org/10.1177/0260106020910907.
17. Gardner, C.D.; Hauser, M.; Gobbo, L.D.; Trepanowski, J.; Rigdon, J.; Ioannidis, J.; King, A.; Desai, M. Neither insulin secretion nor genotype pattern modify 12-month weight loss effects of healthy low-fat vs. healthy low-carbohydrate diets among adults with obesity. *Circulation* **2017**, *135*. https://doi.org/10.1161/circ.135.suppl_1.mp052.

18. Gratz, S.W.; Scobbie, L.; Richardson, A.J.; Zhang, X.; Fyfe, C.; Farquharson, F.M.; Duncan, G.; Filipe, J.; Zhu, W.Y.; Johnstone, A.M.; et al. Comparison of meat versus soya based high-protein diets on faecal microbiota and microbial metabolites. *Proc. Nutr. Soc.* **2020**, *79*, E781. https://doi.org/10.1017/S0029665120007673.
19. Grembi, J.A.; Nguyen, L.H.; Haggerty, T.D.; Gardner, C.D.; Holmes, S.P.; Parsonnet, J. Gut microbiota plasticity is correlated with sustained weight loss on a low-carb or low-fat dietary intervention. *Sci. Rep.* **2020**, *10*, 1405. https://doi.org/10.1038/s41598-020-58000-y.
20. Heianza, Y.; Ma, W.; Sun, D.; Smith, S.R.; Bray, G.A.; Sacks, F.M.; Qi, L. Changes in gut microbiota metabolites and successful weight-loss in response to weight-loss diets: The POUNDS lost trial. *Circulation* **2017**, *136*, A14459.
21. Heianza, Y.; Sun, D.; Ma, W.; Zheng, Y.; Champagne, C.M.; Bray, G.A.; Sacks, F.M.; Qi, L. Gut-microbiome-related LCT genotype and 2-year changes in body composition and fat distribution: The POUNDS Lost Trial. *Int. J. Obes.* **2018**, *42*, 1565–1573. https://doi.org/10.1038/s41366-018-0046-9.
22. Heinsen, F.A.; Fangmann, D.; Müller, N.; Schulte, D.M.; Rühlemann, M.C.; Türk, K.; Settgast, U.; Lieb, W.; Baines, J.F.; Schreiber, S.; et al. Beneficial Effects of a Dietary Weight Loss Intervention on Human Gut Microbiome Diversity and Metabolism Are Not Sustained during Weight Maintenance. *Obes. Facts* **2016**, *9*, 379–391. https://doi.org/10.1159/000449506.
23. Henning, S.M.; Yang, J.; Woo, S.L.; Lee, R.P.; Huang, J.; Rasmusen, A.; Carpenter, C.L.; Thames, G.; Gilbuena, I.; Tseng, C.H.; et al. Hass Avocado Inclusion in a Weight-Loss Diet Supported Weight Loss and Altered Gut Microbiota: A 12-Week Randomized, Parallel-Controlled Trial. *Curr. Dev. Nutr.* **2019**, *3*, nzz068. https://doi.org/10.1093/cdn/nzz068.
24. Hjorth, M.F.; Blædel, T.; Bendtsen, L.Q.; Lorenzen, J.K.; Holm, J.B.; Kiilerich, P.; Roager, H.M.; Kristiansen, K.; Larsen, L.H.; Astrup, A. Prevotella-to-Bacteroides ratio predicts body weight and fat loss success on 24-week diets varying in macronutrient composition and dietary fiber: Results from a post-hoc analysis. *Int. J. Obes.* **2019**, *43*, 149–157. https://doi.org/10.1038/s41366-018-0093-2.
25. Hjorth, M.F.; Christensen, L.; Larsen, T.M.; Roager, H.M.; Krych, L.; Kot, W.; Nielsen, D.S.; Ritz, C.; Astrup, A. Pretreatment Prevotella-to-Bacteroides ratio and salivary amylase gene copy number as prognostic markers for dietary weight loss. *Am. J. Clin. Nutr.* **2020**, *111*, 1079–1086. https://doi.org/10.1093/ajcn/nqaa007.
26. Holt, P.R.; Aleman, J.O.; Bokulich, N.A.; Swann, J.R.; Dannenberg, A.J.; Blaser, M.J.; Hudis, C.A.; Breslow, J. Vlcd-induced rapid weight loss is accompanied by enhanced lipolysis, altered adipose tissues with concomitant changes in the fecal microbiome and plasma metabolome. *Gastroenterology* **2016**, *150*, S9.
27. Hric, I.; Ugrayová, S.; Penesová, A.; Rádiková, Ž.; Kubáňová, L.; Šardzíková, S.; Baranovičová, E.; Klučár, Ľ.; Beke, G.; Grendar, M.; et al. The Efficacy of Short-Term Weight Loss Programs and Consumption of Natural Probiotic Bryndza Cheese on Gut Microbiota Composition in Women. *Nutrients* **2021**, *13*, 1753. https://doi.org/10.3390/nu13061753.
28. Jie, Z.; Yu, X.; Liu, Y.; Sun, L.; Chen, P.; Ding, Q.; Gao, Y.; Zhang, X.; Yu, M.; Liu, Y.; et al. The Baseline Gut Microbiota Directs Dieting-Induced Weight Loss Trajectories. *Gastroenterology* **2021**, *160*, 2029–2042.e2016. https://doi.org/10.1053/j.gastro.2021.01.029.
29. Kahleova, H.; Holubkov, R.; Barnard, N. 506-P: Weight Loss Is Associated with Changes in Gut Microbiome: A Randomized, Crossover Trial Comparing a Mediterranean and a Low-Fat Vegan Diet in Overweight Adults. *Diabetes* **2021**, *70*, N.PAG. https://doi.org/10.2337/db21-506-P.
30. Karl, J.P.; Xueyan, F.; Xiaoxin, W.; Yufeng, Z.; Jian, S.; Chenhong, Z.; Wolfe, B.E.; Saltzman, E.; Liping, Z.; Booth, S.L. Fecal menaquinone profiles of overweight adults are associated with gut microbiota composition during a gut microbiota-targeted dietary intervention. *Am. J. Clin. Nutr.* **2015**, *102*, 84–93. https://doi.org/10.3945/ajcn.115.109496.
31. Kayser, B.D.; Prifti, E.; Lhomme, M.; Belda, E.; Dao, M.C.; Aron-Wisnewsky, J.; Kontush, A.; Zucker, J.D.; Rizkalla, S.W.; Dugail, I.; et al. Elevated serum ceramides are linked with obesity-associated gut dysbiosis and impaired glucose metabolism. *Metabolomics* **2019**, *15*, 140. https://doi.org/10.1007/s11306-019-1596-0.
32. Kong, L.; Wuillemin, P.; Hajduch, F.; Bastard, J.; Fellahi, S.; Basdevant, A.; Zucker, J.; Doré, J.; Rizkalla, S.; Clément, K. Insulinemia and inflammatory markers might predict different responses of obese subjects under the same hypocaloric diet intervention. *Ann. Nutr. Metab.* **2011**, *58*, 75–76. https://doi.org/10.1159/000334393.
33. Kong, L.C.; Hajduch, F.; Wuillemin, P.H.; Bastard, J.P.; Fellahi, S.; Bonnefont-Rousselot, D.; Bittar, R.; Basdevant, A.; Zucker, J.D.; Doré, J.; et al. Plasma insulin and inflammatory markers prior to weight loss can predict dietary responders. *Diabetes* **2011**, *60*, A517. https://doi.org/10.2337/db11-1625-1928.
34. Lee, C.; Florea, L.; Potter, J.; Sears, C.; Durkin, N.; Scudder, M.; Maruthur, N.M.; Schweitzer, M.; Magnuson, T.; Steele, K.; et al. Changes in gut microbiome after medical vs. surgical weight loss in a randomized trial. *Diabetes* **2016**, *65*, A508. https://doi.org/10.2337/db16-1771-2041.
35. Li, X.; Sun, D.; Zhou, T.; Heianza, Y.; Bray, G.; Sacks, F.; Qi, L. Changes in Gut microbiota metabolite TMAO are related to changes in hepatic and visceral fat in weightloss diet interventions: The POUNDS Lost trial. *Circulation* **2019**, *139*. https://doi.org/10.1161/circ.139.suppl_1.MP60.

36. Lin, W.; Lin, B.Y.; Lin, W.; Huang, C. Gut microbiota change after group weight reduction program among obese adults. *Obes. Facts* **2018**, *11*, 289. https://doi.org/10.1159/000489691.
37. Lin, B.Y.; Lin, W.D.; Huang, C.K.; Hsin, M.C.; Lin, W.Y.; Pryor, A.D. Changes of gut microbiota between different weight reduction programs. *Surg. Obes. Relat. Dis.* **2019**, *15*, 749–758. https://doi.org/10.1016/j.soard.2019.01.026.
38. Kong, L.C.; Wuillemin, P.-H.; Bastard, J.-P.; Sokolovska, N.; Gougis, S.; Fellahi, S.; Darakhshan, F.; Bonnefont-Rousselot, D.; Bittar, R.; Doré, J.; et al. Insulin resistance and inflammation predict kinetic body weight changes in response to dietary weight loss and maintenance in overweight and obese subjects by using a Bayesian network approach1-4. *Am. J. Clin. Nutr.* **2013**, *98*, 1385–1394. https://doi.org/10.3945/ajcn.113.058099.
39. Louis, S.; Tappu, R.M.; Damms-Machado, A.; Huson, D.H.; Bischoff, S.C. Characterization of the Gut Microbial Community of Obese Patients Following a Weight-Loss Intervention Using Whole Metagenome Shotgun Sequencing. *PLoS ONE* **2016**, *11*, e0149564. https://doi.org/10.1371/journal.pone.0149564.
40. MacHado, A.D.; Förster-Fromme, K.; Mitra, S.; Friedrich, A.; Kramer, K.; Huson, D.H.; Bischoff, S.C. Competence network obesity: An integrated multi-omics approach to study the guest-host metabolic interaction during restrictive obesity intervention. *Obes. Facts* **2012**, *5*, 4–5. https://doi.org/10.1159/000236230.
41. Mahadzir, M.D.A.; Shyam, S.; Barua, A.; Krishnappa, P.; Ramamurthy, S. Effect of probiotic microbial cell preparation (MCP) on fasting blood glucose, body weight, waist circumference, and faecal short chain fatty acids among overweight Malaysian adults: A pilot randomised controlled trial of 4 weeks. *Malays. J. Nutr.* **2017**, *23*, 329–341.
42. Mueller, N.T.; Differding, M.K.; Maruthur, N.; Juraschek, S.; Miller, E.R.; Yeh, H.C. Effects of metformin and behavioral weight loss on the gut microbiome. *Circulation* **2020**, *141*. https://doi.org/10.1161/circ.141.suppl-1.MP11.
43. Muñiz-Pedrogo, D.A.; Schmidt, B.A.; Jensen, M.D.; Murray, J.A.; Kashyap, P.C.; Nehra, V. Individualized responses to lifestyle interventions can be predicted by gut microbiota. *United Eur. Gastroenterol. J.* **2016**, *4*, A337–A338. https://doi.org/10.1177/2050640616663689.
44. Muñiz-Pedrogo, D.A.; Jensen, M.D.; Van Dyke, C.T.; Murray, J.A.; Woods, J.A.; Chen, J.; Kashyap, P.C.; Nehra, V. Gut Microbial Carbohydrate Metabolism Hinders Weight Loss in Overweight Adults Undergoing Lifestyle Intervention With a Volumetric Diet. *Mayo Clin. Proc.* **2018**, *93*, 1104–1110. https://doi.org/10.1016/j.mayocp.2018.02.019.
45. Muralidharan, J.; Moreno-Indias, I.; Bulló, M.; Lopez, J.V.; Corella, D.; Castañer, O.; Vidal, J.; Atzeni, A.; Fernandez-García, J.C.; Torres-Collado, L.; et al. Effect on gut microbiota of a 1-y lifestyle intervention with Mediterranean diet compared with energy-reduced Mediterranean diet and physical activity promotion: PREDIMED-Plus Study. *Am. J. Clin. Nutr.* **2021**, *114*, 1148–1158. https://doi.org/10.1093/ajcn/nqab150.
46. Nogacka, A.M.; de los Reyes-Gavilán, C.G.; Martínez-Faedo, C.; Ruas-Madiedo, P.; Suarez, A.; Mancabelli, L.; Ventura, M.; Cifuentes, A.; León, C.; Gueimonde, M.; et al. Impact of Extreme Obesity and Diet-Induced Weight Loss on the Fecal Metabolome and Gut Microbiota. *Mol. Nutr. Food Res.* **2020**, *65*, 2000030. https://doi.org/10.1002/mnfr.202000030.
47. Oh, B.; Kim, J.S.; Kweon, M.; Kim, B.S.; Huh, I.S. Six-week Diet Correction for Body Weight Reduction and Its Subsequent Changes of Gut Microbiota: A Case Report. *Clin. Nutr. Res.* **2016**, *5*, 137–140. https://doi.org/10.7762/cnr.2016.5.2.137.
48. Ott, B.; Skurk, T.; Hastreiter, L.; Lagkouvardos, I.; Fischer, S.; Büttner, J.; Kellerer, T.; Clavel, T.; Rychlik, M.; Haller, D.; et al. Effect of caloric restriction on gut permeability, inflammation markers, and fecal microbiota in obese women. *Sci. Rep.* **2017**, *7*, 11955. https://doi.org/10.1038/s41598-017-12109-9.
49. Reimer, R.A.; Willis, H.J.; Tunnicliffe, J.M.; Park, H.; Madsen, K.L.; Soto-Vaca, A. Inulin-type fructans and whey protein both modulate appetite but only fructans alter gut microbiota in adults with overweight/obesity: A randomized controlled trial. *Mol. Nutr. Food Res.* **2017**, *61*, 1700484. https://doi.org/10.1002/mnfr.201700484.
50. Remely, M.; Tesar, I.; Hippe, B.; Gnauer, S.; Rust, P.; Haslberger, A.G. Gut microbiota composition correlates with changes in body fat content due to weight loss. *Benef. Microbes* **2015**, *6*, 431–439. https://doi.org/10.3920/bm2014.0104.
51. Rinott, E.; Youngster, I.; Meir, A.Y.; Tsaban, G.; Kaplan, A.; Zelicha, H.; Rubin, E.; Koren, O.; Shai, I. Autologous fecal microbiota transplantation can retain the metabolic achievements of dietary interventions. *Eur. J. Intern. Med.* **2021**, *92*, 17–23. https://doi.org/10.1016/j.ejim.2021.03.038.
52. Roager, H.M.; Vogt, J.K.; Kristensen, M.; Hansen, L.B.S.; Ibrügger, S.; Mærkedahl, R.B.; Bahl, M.I.; Lind, M.V.; Nielsen, R.L.; Frøkiær, H.; et al. Whole grain-rich diet reduces body weight and systemic low-grade inflammation without inducing major changes of the gut microbiome: A randomised cross-over trial. *Gut* **2019**, *68*, 83–93. https://doi.org/10.1136/gutjnl-2017-314786.
53. Russell, W.R.; Gratz, S.W.; Duncan, S.H.; Holtrop, G.; Ince, J.; Scobbie, L.; Duncan, G.; Johnstone, A.M.; Lobley, G.E.; Wallace, R.J.; et al. High-protein, reduced-carbohydrate weight-loss diets promote metabolite

profiles likely to be detrimental to colonic health. *Am. J. Clin. Nutr.* **2011**, *93*, 1062–1072. https://doi.org/10.3945/ajcn.110.002188.
54. Salonen, A.; Lahti, L.; Salojärvi, J.; Holtrop, G.; Korpela, K.; Duncan, S.H.; Date, P.; Farquharson, F.; Johnstone, A.M.; Lobley, G.E.; et al. Impact of diet and individual variation on intestinal microbiota composition and fermentation products in obese men. *ISME J.* **2014**, *8*, 2218–2230. https://doi.org/10.1038/ismej.2014.63.
55. Sánchez Alcoholado, L.; Gutiérrez Repiso, C.; Alcaide, J.; García Fuentes, E.; Bernal López, R.; Tinahones, F.; Moreno Indias, I. Gut microbiota is differentially affected by distinct weight loss strategies. *Obes. Facts* **2019**, *12*, 193–194. https://doi.org/10.1159/000489691.
56. Siebert, J.C.; Stanislawski, M.A.; Zaman, A.; Ostendorf, D.M.; Konigsberg, I.R.; Jambal, P.; Ir, D.; Bing, K.; Wayland, L.; Scorsone, J.J.; et al. Multiomic Predictors of Short-Term Weight Loss and Clinical Outcomes During a Behavioral-Based Weight Loss Intervention. *Obesity* **2021**, *29*, 859–869. https://doi.org/10.1002/oby.23127.
57. Simões, C.D.; Maukonen, J.; Scott, K.P.; Virtanen, K.A.; Pietiläinen, K.H.; Saarela, M. Impact of a very low-energy diet on the fecal microbiota of obese individuals. *Eur. J. Nutr.* **2014**, *53*, 1421–1429. https://doi.org/10.1007/s00394-013-0645-0.
58. von Schwartzenberg, R.J.; Bisanz, J.E.; Lyalina, S.; Spanogiannopoulos, P.; Ang, Q.Y.; Cai, J.; Dickmann, S.; Friedrich, M.; Liu, S.Y.; Collins, S.L.; et al. Caloric restriction disrupts the microbiota and colonization resistance. *Nature* **2021**, *595*, 272–277. https://doi.org/10.1038/s41586-021-03663-4.
59. Yoriko, H.; Dianjianyi, S.; Smith, S.R.; Bray, G.A.; Sacks, F.M.; Lu, Q.; Heianza, Y.; Sun, D.; Qi, L. Changes in Gut Microbiota-Related Metabolites and Long-term Successful Weight Loss in Response to Weight-Loss Diets: The POUNDS Lost Trial. *Diabetes Care* **2018**, *41*, 413–419. https://doi.org/10.2337/dc17-2108.
60. Yuan, W.; Lu, W.; Wang, H.; Wu, W.; Zhou, Q.; Chen, Y.; Lee, Y.K.; Zhao, J.; Zhang, H.; Chen, W. A multiphase dietetic protocol incorporating an improved ketogenic diet enhances weight loss and alters the gut microbiome of obese people. *Int. J. Food Sci. Nutr.* **2021**, *73*, 238–250. https://doi.org/10.1080/09637486.2021.1960957.
61. Zhou, T.; Heianza, Y.; Chen, Y.; Li, X.; Sun, D.; DiDonato, J.A.; Pei, X.; LeBoff, M.S.; Bray, G.A.; Sacks, F.M.; et al. Circulating Gut Microbiota Metabolite Trimethylamine N-Oxide (TMAO) and Changes in Bone Density in Response to Weight Loss Diets: The POUNDS Lost Trial. *Diabetes Care* **2019**, *42*, 1365–1371. https://doi.org/10.2337/dc19-0134.
62. Zolotarevsky, M.V.; Zolotarevsky, E.; DeBenedet, A.; Duda, J.; Velarde, M.; Lyons, D.; Montagano, J.; Gunaratnam, N.T. A multifacted approach to weight loss is effective in obese NASH and GERD patients in a community GI practice: An interim analysis. *Gastroenterology* **2018**, *154*, S-431. https://doi.org/10.1016/S0016-5085(18)31706-2.
63. Zou, H.; Wang, D.; Ren, H.; Cai, K.; Chen, P.; Fang, C.; Shi, Z.; Zhang, P.; Wang, J.; Yang, H.; et al. Effect of Caloric Restriction on BMI, Gut Microbiota, and Blood Amino Acid Levels in Non-Obese Adults. *Nutrients* **2020**, *12*, 631. https://doi.org/10.3390/nu12030631.
